# 3D Nanocomposite with High Aspect Ratio Based on Polyaniline Decorated with Silver NPs: Synthesis and Application as Electrochemical Glucose Sensor

**DOI:** 10.3390/nano13061002

**Published:** 2023-03-10

**Authors:** Anna A. Vasileva, Daria V. Mamonova, Vladimir Mikhailovskii, Yuri V. Petrov, Yana G. Toropova, Ilya E. Kolesnikov, Gerd Leuchs, Alina A. Manshina

**Affiliations:** 1 Institute of Chemistry, Saint-Petersburg State University, Ulyanovskaya st. 5, Saint-Petersburg 198504, Russia; 2 Interdisciplinary Resource Center for Nanotechnology, Research Park, Saint-Petersburg State University, Ulyanovskaya 1, Saint-Petersburg 198504, Russia; 3 Department of Physics, Saint-Petersburg State University, Ulyanovskaya st. 3, Saint-Petersburg 198504, Russia; 4 Almazov National Medical Research Centre, Akkuratova st. 2, Saint-Petersburg 197341, Russia; 5 Center for Optical and Laser Materials Research, Saint-Petersburg State University, Ulyanovskaya 5, Saint-Petersburg 198504, Russia; 6 Max Planck Institute for the Science of Light, Staudtstr. 2, 91058 Erlangen, Germany; 7 Department of Physics, Friedrich-Alexander-Universität Erlangen-Nürnberg, Staudtstr. 7/B2, 91058 Erlangen, Germany

**Keywords:** glucose sensor, laser-induced deposition, polyaniline, anodic aluminum oxide

## Abstract

In this paper, we present a new methodology for creating 3D ordered porous nanocomposites based on anodic aluminum oxide template with polyaniline (PANI) and silver NPs. The approach includes in situ synthesis of polyaniline on templates of anodic aluminum oxide nanomembranes and laser-induced deposition (LID) of Ag NPs directly on the pore walls. The proposed method allows for the formation of structures with a high aspect ratio of the pores, topological ordering and uniformity of properties throughout the sample, and a high specific surface area. For the developed structures, we demonstrated their effectiveness as non-enzymatic electrochemical sensors on glucose in a concentration range crucial for medical applications. The obtained systems possess high potential for miniaturization and were applied to glucose detection in real objects—laboratory rat blood plasma.

## 1. Introduction

Accurate and fast glucose detection in small volumes of biological analytes has remained a vital challenge for many years. Diabetes is one of the life-threatening diseases of mankind. However, regular monitoring of blood glucose can prevent hypo- and hyperglycemia, thereby prolonging the life of diabetic patients. Small glucometer devices make life easier; however, they require the combination of several important characteristics. Simultaneous requirements are miniaturization, accuracy, operation speed in small volumes of the analyte (a drop of the patient’s blood). Ensuring the listed important characteristics stimulates scientists in the search for new solutions.

Thus, the quest for easy-to-use patient-friendly detection of glucose has been most challenging for science in recent decades. Different routes are explored among which non-invasive glucose detection based, e.g., on sensing with acoustic and light waves, has particular appeal. While there have been promising advances [1,2], the breakthrough is still to come. That is why electrochemical sensors are still considered as most promising [3]. The latest, fourth generation of electrochemical glucose sensors is based on a non-enzymatic approach. Non-enzymatic sensors are characterized by direct electrocatalytic oxidation of glucose on the electrode surface that provides faster and more accurate glucose detection with higher reproducibility and stability during a prolonged operation.

Non-enzymatic glucose detection was demonstrated for various electrode materials such as mono- and multimetallic nanostructures [4,5,6,7,8,9], carbon-based nanocomposites [10,11,12,13], and conductive polymers (CPs) [14,15,16,17,18]. Improving the analytical signal for non-enzymatic glucose detection was demonstrated for composite materials combining conductive polymers and metallic nanoparticles (MNPs) [15,19,20,21,22]. Furthermore, nanocomposites based on CP—polyaniline (PANI) present a special group promising for the creation of small glucometer devices. The main advantages of PANI as a material for glucometer devices are (i) high electron conductivity (thus providing total electrochemical potency), (ii) a large surface area of PANI due to its porous structure (it provides an increased surface for MNPs sites active in electrocatalysis), (iii) chemical stability (this allows various chemical approaches for the creation of PANI-based nanocomposites), (iv) biocompatibility (promising for sensors as implantable devices with tolerance to body fluids or tissues), (v) flexibility—potency for nanocomposites with 3D architecture (it provides the miniaturization option). All the listed features have attracted the attention of researchers to PANI as an important component of nanocomposites for glucose electrochemical sensors [23,24].

In spite of the advantages of PANI-based materials demonstrated for glucose sensing, there are still problems to be solved for their implementation in real applications. To make materials promising for electrochemical glucose detection, one should keep in mind the potential of the studied systems with respect to miniaturization and integration. Moreover, for adequate device operation, a controllable morphology with a highly electro-catalytically active area of the sample is essential. However, most of the reported systems are disordered matrices with catalytically active NPs on the surfaces of wires, disordered pores, etc. [25,26,27], leaving no possibility for morphology control and reproducibility.

Here we present the synthesis of nanocomposites based on PANI with incorporated Ag nanoparticles. The suggested approach allows the creation of 3D-ordered porous nanocomposites with a high aspect ratio of the pores, topological ordering, uniformity of properties throughout the sample and a large specific surface area. Ordered 3D architecture of the PANI/Ag nanocomposite was ensured by a template of anodic aluminum oxide (AAO); the PANI layer on AAO pores was obtained by developed in situ oxidative polymerization of aniline; Ag NPs on the inner surface of the PANI-covered AAO pores were synthesized by laser-induced deposition (LID). The important LID peculiarity is NPs nucleation and growth directly on the substrate surface, thus providing good NPs adhesion. The presented 3D PANI/Ag nanocomposite was studied as an electrode in the electrochemical reaction of glucose oxidation. It was found that the 3D PANI/Ag nanocomposite acts as a potentiometric sensor on glucose in a range of glucose concentrations for real medical diagnostic applications (5–15 mM). We demonstrated the applicability of such structures for glucose detection in Ringer’s solutions and in the blood plasma of a laboratory rat. In such a way, the suggested 3D ordered porous PANI/Ag nanocomposites can be considered as new agents of non-enzymatic electrochemical glucose sensors. Another competitive advantage of the 3D PANI/Ag nanocomposite is the potency of a miniature device design for the study of microliter volumes of analytes.

## 2. Experimental Details

### 2.1. Materials and Methods

Aluminum plates (99.99%), were used for AAO membranes synthesis. CuCl_2_ (>98% wt%), H_3_PO_4_ (84% wt%), HClO_4_ (72% wt%), HCl (38% wt%), CH_4_O, and C_2_H_6_O (spectroscopic grade) were purchased from Reachem (Moscow, Russia) and used without additional purification. For the PANI synthesis, C_6_H_5_NH_2_ was preliminarily purified using a standard procedure [28]; (NH_4_)_2_S_2_O_8_ (highly purified) was stored in darkness in the presence of desiccant and used as received. For solutions preparation, bidistilled water was used. As a precursor for laser-induced deposition, silver salt C_6_H_5_COOAg (containing Ag 47.1 wt%) from Alfa Aesar (Haverhill, MA, USA) was used. For the electrochemistry, Ringer’s solution was prepared (0.11 M NaCl, 0.06 M KCl, 0.02 M CaCl_2_) with different concentrations of glucose. Reagents (highly purified) were purchased from Reachem (Moscow, Russia) and used as received. Samples of terminal blood plasma from laboratory rats were used as a biological analyte for glucose detection. The samples were provided by the V.A. Almazov Scientific Research Center, Saint-Petersburg, Russia. After collection, the plasma was frozen and stored frozen at −80 °C. Defrosting was performed just before electrochemical measurements.

### 2.2. Synthesis of Composites

First, highly ordered membranes of anodic aluminum oxide were prepared to be used as 3D templates. For the preparation, aluminum plates were electropolished in precooled ethanol solution of perchloric acid (volume ratio 3:1), applying 20 V during 15 min. Membranes were then synthesized in a two-step anodization procedure. The anodization was performed in 0.5% H_3_PO_4_ solution, applying 180 V; the temperature in the chamber was maintained at 2 °C. The first step of anodization was carried out over 24 h. The obtained porous structure was then removed by exposition of samples in aqueous solution of 0.3 M potassium dichromate and 10% of phosphoric acid for 24 h at 45 °C. As a result, an ordered aluminum surface was formed. The second step of anodization was subsequently carried out in the conditions of 1% H_3_PO_4_, 180 V, +2 °C for 8 h. Finally, the back-side aluminum was removed from the sample by 0.7 M CuCl_2_ in 10% HCl solution treatment and the barrier oxide layer was removed by sample exposition in 10% H_3_PO_4_ at 45 °C for 40 min. Obtained ordered AAO membranes were used as templates for in situ polyaniline synthesis.

For the polyaniline synthesis, aqueous solutions of aniline hydrochloride (0.04 M) and ammonium persulfate (0.05 M) were prepared and stored for 1 h at a temperature of +2 °C. The volume of each solution was 5 mL. The substrate was placed into a beaker and precooled. The synthesis itself was performed in a chamber at a temperature of +2 °C. Aniline hydrochloride and ammonium persulfate solutions were poured into the beaker, shaken for several seconds and left for diffusion into AAO pores and polymerization for 1 h. The sample was then removed from the reaction zone and dried in an airflow. Subsequently, a part of the samples was placed into a spin-coater and rotated for 10 min at 400 rpm. Obtained AAO/PANI structures were used for subsequent laser-induced deposition of silver NPs.

For laser-induced deposition of silver NPs, the AAO/PANI sample was placed into a precursor solution of silver benzoate in methanol (0.4 mM) and stored in darkness for 3 h. Then the sample was removed from the solution and placed under continuous wave laser irradiation with a wavelength of 266 nm. The laser beam was unfocused, and the diameter of the laser spot was 1 cm^2^. The irradiation power density was 15 mW/cm^2^. The exposition was carried out during 40 min. After the LID procedure, AAO/PANI/Ag samples were ready for further operations.

### 2.3. Samples Characterization

The polyaniline structure was studied by Raman spectroscopy using a Senterra (Bruker, Billerica, MA, USA) setup. The Raman spectra were excited by a 785-nm solid-state laser (1 mW power) using a 20× objective with a 200 s acquisition time, and the spectra were collected four times. The morphology of AAO/PANI structures was studied by scanning electron microscopy (SEM) using a Zeiss Merlin microscope (from Karl Zeiss, Oberkochen, Germany) equipped with a field emission cathode, a GEMINI-II electron-optics column, and an INCAx-act energy dispersive X-ray spectrometer (EDX), all in an oil-free vacuum system (Oxford Instruments, Abingdon, UK). The measurements were performed using a secondary electrons detector. The sample cross-section view was studied.

Results of the laser-induced deposition were studied by Raman spectroscopy. The Raman spectra were excited by a 532-nm solid-state laser (10 mW power) using a 10× objective with an 80 s acquisition time, and the spectra were collected twice.

For the study of the AAO/PANI/Ag structures morphology, SEM measurements were applied. The data were collected using a secondary electrons detector and back-scattered electron detectors from the top and cross-view of the sample. Additionally, EDX scanning across the sample was performed.

### 2.4. Electrochemical Measurements

The electrochemical characterization was performed in a three-electrode cell with a sample as a working electrode, the Ag/AgCl(KCl_3.5M_) electrode as a reference and a platinum mesh as a counter electrode. The measurements were performed in Ringer’s solution (0.11 M NaCl, 0.06 M KCl, 0.02 M CaCl_2_) with different concentrations of glucose. The pH value was corrected to be 7 ± 0.1 by addition of the NaOH solution. Cyclic voltammetry (CV) and impedance spectroscopy (EIS) were used for the characterization. Measurements were carried out with a potentiostat/galvanostat Gamry Reference 600 (Gamry Instruments, Warminster, PA, USA). The cyclic voltammetry measurements were recorded at Vscan = 50 mV/s; the third cycles are presented. The impedance spectroscopy measurements were performed across a range from 100 kHz to 0.01 Hz in potentiostatic mode with the application of +0.5 V potential. The amplitude of the applied sinusoidal voltage was ±10 mV.

In the next stage of the study, glucose detection by CV measurements in rat blood plasma as a real biological sample was carried out. For this, the electrochemical cell was adapted for small analyte volumes (see Supplementary Materials). The performed modernization made it possible to carry out measurements in the electrolyte volume of 100 µL.

Samples of terminal blood plasma from the laboratory rats (mature male SPF Wistar rats weighing 283 ± 22 g) were used as a biological analyte for glucose detection. Animals were housed in a barrier-type facility in standard environmental conditions: 12 h light/dark cycle and standard temperature and humidity; and were administered food and water ad libitum. The animals study protocol was approved by an institutional animal care and use committee of the Centre for Experimental Biomodelling, Institute of Experimental Medicine, Almazov National Medical Research Centre, Ministry of Health of the Russian Federation (П3N°16-7, 19 August 2016). Blood samples were taken from the posterior vena cava. After collection, the plasma was frozen and stored frozen at −80 °C.

Additionally, in blood plasma samples the glucose level was analyzed. The analysis was performed using ChemWell 2910 Combi automatic bio-chemical and enzyme-linked immunosorbent assay (ELISA) analyzer (Awareness Technology, SW Martin Hwy, Palm City, FL, USA) and a commercial kit, according to the manufacturer’s instructions. The results obtained were recorded using the Windows-based ChemWell Manager software (NEOGEN Corporation, 620 Lesher Place, Lansing, MI, USA). The provided samples of the blood plasma were placed in the cell without additional sample preparation.

## 3. Results and Discussion

### 3.1. Synthesis and Characterization of AAO/PANI/Ag Nanocomposite

**Figure 1:**
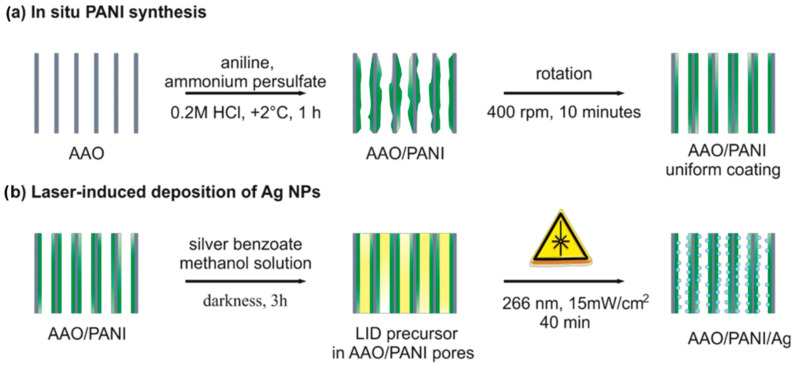
Schematic presentation of AAO/PANI/Ag nanocomposite synthesis procedure; (**a**) PANI synthesis on AAO template; (**b**) laser-induced deposition of Ag NPs on 3D PANI.

To prepare the PANI/Ag nanocomposite on AAO membranes, a two-step approach was chosen (Figure 1).

In situ PANI synthesis on AAO (Figure 1a) was followed by laser-induced deposition of catalytically active Ag NPs (Figure 1b). The AAO templates with pores with a diameter of d = 338 ± 43 nm and a length of L = 22 ± 0.8 µm were used for the study.

#### 3.1.1. PANI Synthesis on AAO Template

According to modern understanding [29], the oxidative polymerization of aniline with PANI formation is realized in several stages: nucleation (formation of phenazine intermediates, their sorption on the substrate and agglomeration) and chains growth (by the reactions on side groups). After the start of the chains growth, the nucleation stage is inhibited. That is why if a substrate is placed in the reaction zone when the nucleation stage has been completed, no polymer formation on the substrate can be observed [30]. Taking this into account, polymer formation on the pore walls of AAO templates with a high L/D ratio is possible only if mass transport of precursors into the pores takes place during the nucleation period. However, in the presence of a template, the duration of the nucleation stage is decreased [31]. Additionally, it is necessary to note that the structure of the obtained polymer is greatly dependent on the synthesis parameters. For electrochemical applications, only polyaniline in the conductive form of emeraldine salt is useful. These circumstances make the formation of PANI coating on the pore walls of AAO templates a great challenge. Utilizing classical UIPAC methodology [32] for the synthesis of PANI in emeraldine form in the case of AAO templates leads to polymer formation on the membrane surface, while the pores remain empty. This is a result of the fact that the duration of the nucleation period is not sufficient to mas transport inside the pores. Thus, the goal of our studies was to increase the nucleation period. For this, the temperature of the synthesis and the precursors concentrations were decreased. The most successful experiments were performed at 2 °C, and with concentrations of monomer and oxidant of 0.04 M and 0.05 M correspondingly (that is, five times lower than the concentrations recommended by IUPAC).

**Figure 2:**
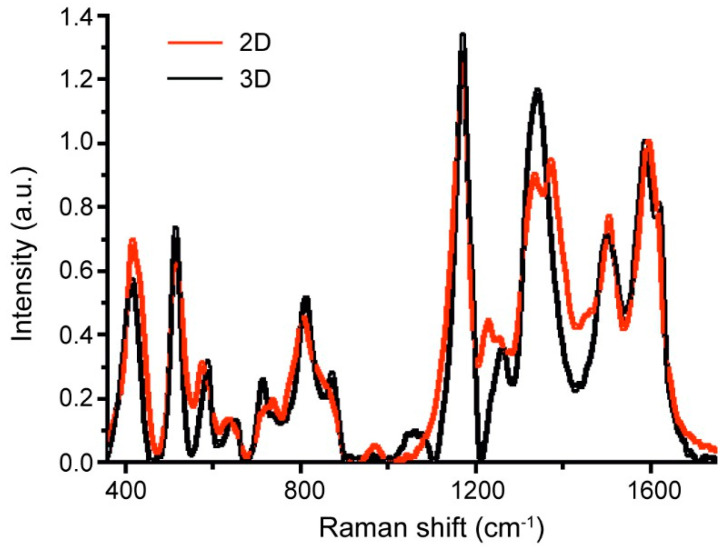
Raman spectra of PANI on 2D and 3D substrates (cover slips and AAO membranes, respectively).

As far as the PANI synthesis conditions on AAO 3D templates significantly differ from IUPAC methodology, to confirm PANI formation in emeraldine form we additionally obtained emeraldine films on planar 2D substrates (cover glasses) for comparison. The polymer structure was studied by Raman spectroscopy; the results are presented in Figure 2.

In accordance with Raman spectra, the structure of the PANI on the 3D substrate is close to the structure on the planar substrate. In both cases, PANI in the form of emeraldine salt was obtained. This is proved by the presence of a 1170 cm^−1^ peak (bending vibrations with hydrogen in ring structures) [33], peaks in a range of 1300–1400 cm^−1^ (stretching modes in C-N^+•^, associated with polarons) [34], 1505 cm^−1^ (stretching modes of C=N bands in quinoid fragments) [35], as well as a peak at 1590 cm^−1^ with a shoulder at 1618 cm^−1^ (stretching modes of C=C bands in quinoid and benzenoid fragments, respectively) [34,35]. However, the difference between 2D and 3D structures was observed in the spectral region of 1300–1400 cm^−1^. In the case of 2D architecture, two well-resolved peaks were observed. These peaks are related to the delocalized charge—polaron (1340 cm^−1^) and the localized charge—bipolaron (1380 cm^−1^). In the case of 3D architecture, only one peak that shifted to 1340 cm^−1^ was observed. This indicates a higher charge delocalization for the PANI with 3D architecture than for the PANI on planar substrates that should provide more effective charge transport in a 3D system [36].

**Figure 3:**
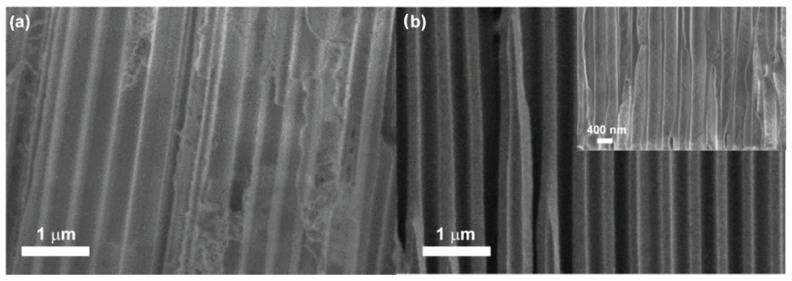
SEM data for AAO/PANI with 3D architecture obtained (**a**) without centrifugation; (**b**) with centrifugation.

The morphology of PANI structures determines the sample surface area that is of a great importance for further electrochemical applications. At first, the samples were characterized by SEM directly after removal from the polymerization zone (Figure 3a). SEM characterization of PANI synthesized on AAO template demonstrated the formation of polymer layer along the pores. However, PANI coating was found to be inhomogeneous with alternating empty areas and areas completely filled with polymer. To improve the morphology, the synthetic procedure was supplemented with a centrifugation step. During the stage of the polymer chains growth, the sample was removed from the reaction zone, placed into a spin-coating setup, and rotated for 10 min at 400 r/min. This approach allows us to obtain a PANI continuous coating with a lower thickness (Figure 3b).

As can be seen from the insert pn Figure 3b, formation of an uniform PANI coating of the inner surface of the AAO pores was achieved. Demonstrated AAO/PANI samples were decorated with silver NPs by laser-induced deposition.

#### 3.1.2. AAO/PANI Decoration with Ag NPs

**Figure 4:**
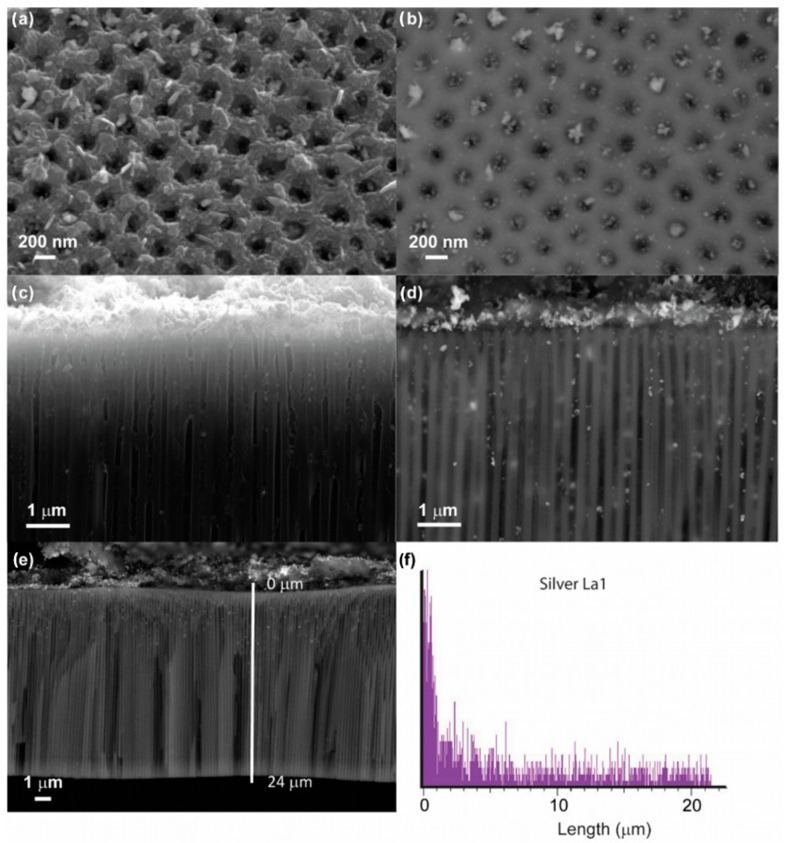
Sample AAO/PANI/Ag; (**a**) SEM data, top view, secondary electrons detector; (**b**) SEM data, top view, backscattered electrons detector; (**c**) SEM data, cross view, secondary electrons detector; (**d**) SEM data, cross view, backscattered electrons detector; (**e**); (**f**) EDX signal of silver distribution along the line.

The important peculiarity of systems based on chosen AAO templates is the high aspect ratio L/d ~ 65. Due to this, it is possible to create the systems with a large electrocatalytically active surface area with a small visible area. A high electrocatalytical response (provided by a large specific surface area) in combination with a small visible area gives an opportunity for miniaturization of final devices. Furthermore, NPs are known to demonstrate high electrocatalytic activity [37]; this is why the loading of high-aspect ratio systems with NPs is very attractive. However, known methods of wet chemistry are useful for the decoration of high-aspect structures of various opal-type or nanowires morphologies [38,39,40,41]. While for inverted opal-type structures (such as AAO), there are no approaches that provide satisfactory coating parameters. Laser-induced deposition is one of the most promising among other methods for the decoration of structures of a complex topology [42]. Moreover, it allows precise control of deposited NPs composition, size, coating density, and localization area [43,44,45,46]. Other important advantages of LID are (i) that nucleation and growth of NPs takes place directly on the laser-affected area of the substrate thus providing good adhesion; (ii) that LID is realized at a low laser intensity, allowing the formation of NPs on polymer substrates [47]. The general scheme of LID of Ag NPs on the 3D AAO/PANI system is presented in Figure 1b and the experimental details are described in Section 2.2. As the PANI can be potentially destroyed by laser irradiation, optimization of the laser intensity was carried out. The optimal laser intensity was found to be 15 mW/cm^2^ as it provides both substrate preservation and efficient formation of Ag NPs. The results of the laser-induced deposition of Ag NPs on the 3D AAO/PANI system are shown in Figure 4.

Figure 4a,b shows SEM images of AAO/PANI/Ag from the top of the sample. The images were obtained using different detectors, a—a secondary electrons detector, b—a backscattered electrons detector; images were taken from the same area. When using a secondary electrons detector, both the polyaniline component and silver nanoparticles can be observed. The use of a backscattered electrons detector makes it possible to observe only heavier silver nanoparticles and the AAO template, while the polymer phase is not detected. Thus, a comparison of the data obtained with different detectors proves the presence of both phases and demonstrates their distribution. Moreover, in the image Figure 4d obtained with the backscattered electrons detector, one can also observe additional diffuse signals from silver NPs located inside pores in deeper areas relative to the cut surface, which proves the formation of NPs over the entire volume of the sample. Figure 4f shows the EDX signal of silver detected in the scanning regime along the line (Figure 4e). One can see that the Ag signal is closer to the substrate surface; nevertheless, silver distribution along the whole pores length is clear. In addition, it is proved by the presence of a silver signal in the EDX spectra recorded from the backside of the sample (see Figure S3).

#### 3.1.3. Mechanism of Ag NPs Formation during the LID Process

**Figure 5:**
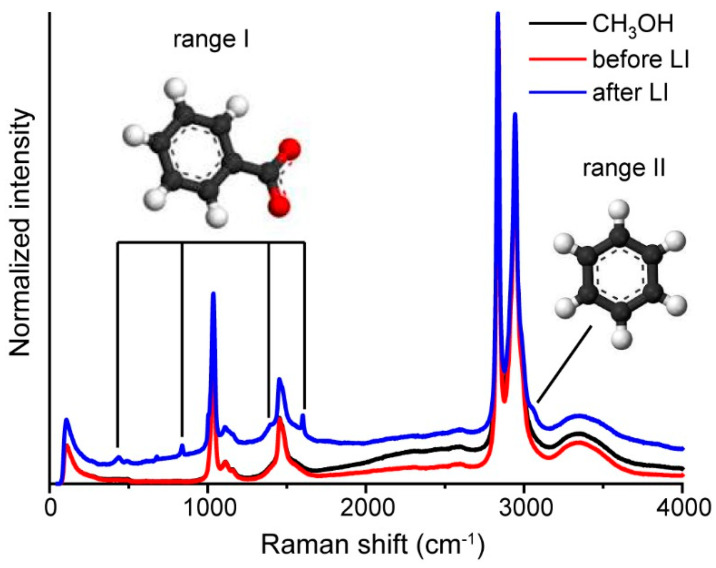
Raman spectra of methanol (black), silver benzoate solution in methanol before exposition under laser irradiation (red), and after exposition under laser irradiation (blue).

To uncover the mechanism of Ag NPs formation during the LID process from the silver benzoate methanol solution, additional experiments were carried out. The silver benzoate methanol solution was placed into a spectrophotometric cuvette and closed with a quartz cover to avoid solvent evaporation during exposition under laser irradiation. Irradiation of the solution volume was then performed. Raman spectra were recorded for the methanol and silver benzoate solution before and after irradiation. The data are presented in Figure 5.

Raman spectra of the methanol and the methanol solution of silver benzoate before laser irradiation are characterized by typical methanol peaks at 3360 cm^−1^ (stretching mode of OH group), 2943 cm^−1^ and 2834 cm^−1^ (stretching mode of C-H), 1454 cm^−1^ (bending mode of C-H) and 1034 cm^−1^ (stretching mode of C-O) [48]. The Raman spectrum of the silver benzoate methanol solution after laser irradiation demonstrates new peaks at 431 cm^−1^ and 836 cm^−1^, (ν_s_ C-C of benzene ring and δ(COO^−^) correspondingly); a shoulder at 1391 cm^−1^, (ν_s_(COO^−^)), peak at 1601cm^−1^ (ν_s_ C-C in benzene ring) (Figure 5, range I) and shoulder at 3067 cm^−1^ (Figure 5, range II). The listed peaks (except for the shoulder at 3067 cm^−1^), are typical for benzoic acid [49,50]. The appearance of new peaks in the spectrum may be explained by the effect of the surface-enhanced Raman scattering on silver NPs formed during irradiation.

The band at 3067 cm^−1^ can be associated with a symmetric stretching mode of C-H vibrations in benzene rings [51,52]. In the Raman spectra of benzoic acid, these vibrations appeared as the band with a maximum at 3085 cm^−1^ [53]. The difference between positions of the C-H peak in benzene (3067 cm^−1^) and benzoic acid (3085 cm^−1^) are explained by the effect of the substitute—carboxylic group. Because the other detected peaks are not shifted in comparison with the peaks of benzoic acid, we can assume that the 3067 cm^−1^ band is related with benzene which may appear as a photodegradation product during the LID process.

This assumption is supported by electron paramagnetic resonance spectroscopy [54]. In this investigation, during UV irradiation of the silver benzoate isopropanol solution, the reaction of benzoate decarboxylation with the formation of phenyl radical takes place. his radical may then interact with a solvent or benzoic acid anion. In the last case, adduct and aqueous electron formation occurs. Taking this process into consideration, we can assume the same processes happens during the LID and the originated electrons taking part in silver ions reduction.

### 3.2. Electrochemical Glucose Detection on 3D AAO/PANI/Ag Nanocomposite

**Figure 6:**
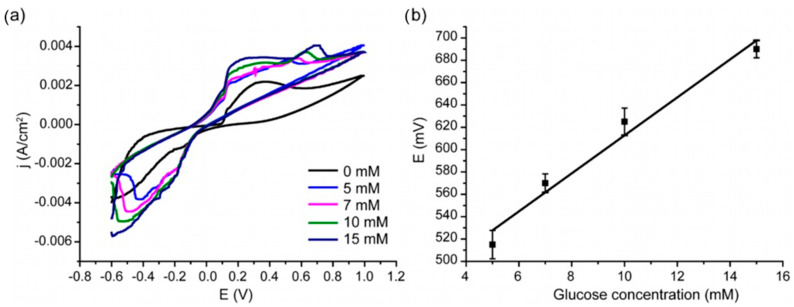
(**a**) CVA measured in the presence of different concentrations of glucose, third cycle; (**b**) calibration curve.

The next stage of the study was devoted to a demonstration of the potency of AAO/PANI/Ag structures to glucose detection. The given current densities were calculated based on the visible area of the sample. Measurements were carried out in the absence and presence of glucose. Figure 6a shows CV data obtained for glucose concentrations close to physiological values (5–15 mM). The data presented vs. the Ag/AgCl electrode.

There are several cathodic and anodic peaks on the presented CVA. A pair of peaks at +0.13 V (anodic curve) and −0.11 V (cathodic curve) indicate the presence of silver NPs in the system. To prove this judgement, additional experiments on laser-induced deposition of silver NPs on the graphite electrode with subsequent measurement of cyclic voltammetry were carried out. It was found that the peaks at +0.13 V and −0.11 V appear in the absence of PANI but in the presence of silver NPs (see Supporting Information, Figure S2).

Glucose was then added to the electrolyte (5 mM concentration) and an anodic peak was found to appear at +0.515 V. With the increase in glucose concentration, the peak shifts to +0.690 V. One of the possible explanations of this observation is a local change in pH that affects the electrochemical response of polyaniline. This effect has been described in the literature [55], but is considered of little applicability for analytical purposes due to the rapid establishment of an equilibrium with the electrolyte. However, in the case of systems with 3D architecture based on AAO, which provide a high aspect ratio of pore length to pore diameter, this effect may be significant. On the other hand, a shift in potential with an increasing glucose concentration is observed on silver nanostructures [56]. That is why for the studied 3D nanocomposite, both effects can be considered as analytic signals for glucose detection. Thus, a 3D AAO/PANI/Ag nanocomposite can be used as a potentiometric sensor for glucose. The corresponding calibration curve is shown in Figure 6b. A linear dependence of the potential on the glucose concentration is observed in the selected concentration range. These concentrations correspond to the physiological range in the blood plasma. The sensor sensitivity is 17 ± 2 mV/mM and limit of detection = 0.35 mM. It is necessary to note that the structure under investigation acts as a potentiometric sensor while a wide range of PANI-based sensors are amperometric ones [3]. Obtained systems demonstrate a linear response in a range of glucose concentrations actual for real medical diagnostic applications while most of studies are devoted to achieving a low limit of detection [57,58,59,60,61].

**Figure 7:**
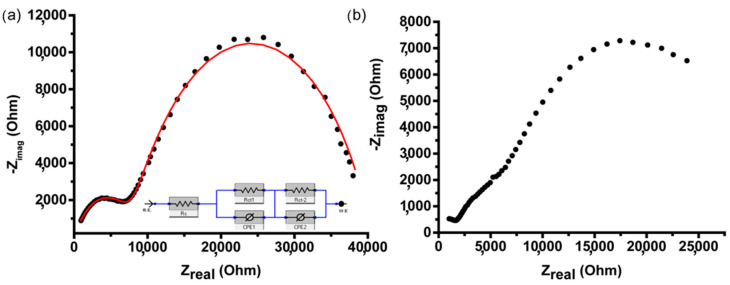
Impedance spectroscopy data. (**a**) In the absence of glucose, equivalent circuit inserted; (**b**) in the presence of 5 mM glucose.

To uncover the nature of the analytical signal observed by cyclic voltammetry, the impedance spectroscopy data were recorded in the absence and presence of glucose in the system. The results are presented in Figure 7.

Figure 7a shows the hodograph obtained in the absence of glucose in the system and reflects the processes occurring in the 3D Ag-PANI nanocomposite itself. Based on the presented data, it can be concluded that two processes of charge transfer are realized. We can assume that one of the processes (which corresponds to the higher-frequency area of the hodograph) is associated with the charge transfer within the polymer component, and the second (the lower-frequency part of the hodograph) is associated with the charge transfer from silver NPs to the polymer. The data were fitted with an equivalent circuit in which two constant phase elements were included. The probable interpretation of them may be related to the high impact of heterogeneity on the phase boundary (1) between the electrolyte and NPs as well as the phase boundary (2) between the electrolyte and polymer, caused by high porosity of the system. The Rct(1) is 325 Ohm and Rct(2) is 791 Ohm while the R(solution) is 49 Ohm. It should be noted that there was no impact from the diffusion. This means that the electrode reaction is the limiting stage. When glucose is added to the system, the pattern in the low-frequency region is changed (see Figure 7b). This indicates that silver NPs are responsible for the analytic signal in the procedure of glucose detection. In the presence of glucose, the equivalent circuit had elements out of physical meaning; this is why it was not included in the discussion.

**Figure 8:**
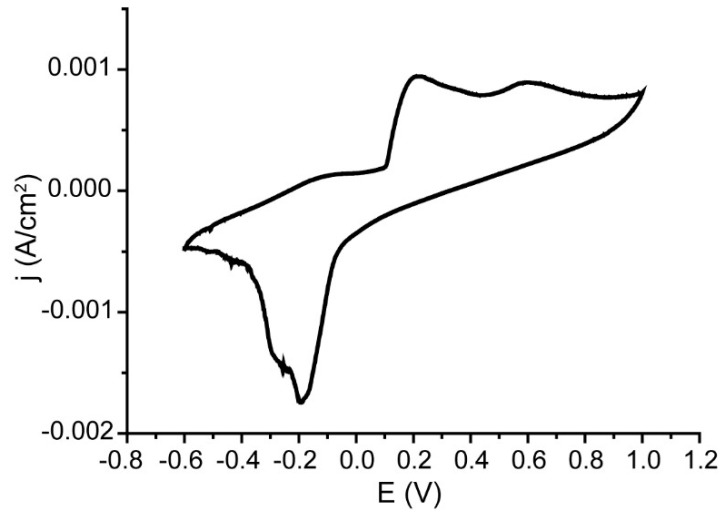
CV for glucose detection in rat blood plasma on AAO/PANI/Ag nanocomposite.

It is necessary to note that the visible area of the working electrode of 3D AAO/PANI/Ag nanocomposite was just 0.07 cm^2^. Thus, such samples demonstrate their miniaturization potential which is critically recommended for the construction of real devices. In the next step of the investigation, the potency of 3D AAO/PANI/Ag nanocomposite as electrochemical glucose sensors for real samples was demonstrated. For this, glucose measurements were carried out using blood plasma from laboratory rats as electrolyte. To perform these measurements, the electrochemical cell with a small volume was constructed (see Figure S1 and description). The volume of the analyzed blood plasma sample was 100 µL. Figure 8 shows CV data recorded in the rat blood plasma electrolyte.

According to the calibration curve (Figure 7b), the concentration of glucose in the studied blood plasma samples is 9.26 ± 0.31 mM. According to the data obtained by biochemical measurements (Section 2.4), the glucose level in the sample was 8.2 mM. The study of the plasma sample using a medical glucometer Accu-Check Active yielded a value of 12 mM. Thus, created 3D AAO/PANI/Ag nanocomposite structures demonstrated their effectiveness as sensors on glucose in real biological samples.

## 4. Conclusions

In this research, we present a unique approach to the synthesis of electrocatalytically active nanostructures with a large specific surface area and the uniformity of properties throughout the sample thanks to the topological ordering. The systems are based on structured templates of anodic aluminum oxide with a high aspect ratio of pores and a high structural order. The templates were covered by uniform layers of polyaniline with embedded catalytically active silver NPs. The synthesis includes two main steps. The first is the developed in situ oxidative aniline polymerization directly on the inner pore walls of the AAO. The obtained coating was uniform, continuous and consisted of polyaniline in the form of emeraldine salt. The second step was the laser-induced deposition of silver NPs on the inner walls of the AAO/PANI system. This approach allowed the synthesis of NPs on the structures with a high aspect ratio and provided good NPs adhesion, as NPs nucleation and growth take place directly on the polyaniline layer.

The proposed approach allowed the creation of AAO/PANI/Ag systems, which demonstrated applicability for electrochemical glucose detection. It was demonstrated that the systems act as a potentiometric sensor on glucose in a range of glucose concentrations actual for biomedical purposes (5–15 mM). In addition, it was found that a charge transfer with the participation of silver NPs is responsible for glucose detection.

The investigated systems successfully demonstrated the operability for glucose detection in real biological objects—the blood plasma of laboratory rats. As a consequence of the high aspect ratio of the system, obtained structures exhibit a large electroactive surface area with a small visible surface area (0.07 cm^2^). Thus, the AAO/PANI/Ag systems demonstrated the potential for miniaturization and application in real biological objects: the volume of the analyzed sample was 100 µL.

The current research achieved several goals: the development of synthetic procedures for obtaining ordered nanocomposite structures, glucose detection on the synthesized structures and demonstration of applicability for biological samples. Thus, the study may make a push for evolution in all the listed areas.

## Data Availability

Data will be available by request.

## Supplementary Materials

The following supporting information can be downloaded at: https://www.mdpi.com/article/10.3390/nano13061002/s1, Figure S1 (a) photo of the detail with counting electrode; (b) scheme of electrochemical cell; Figure S2 CV data for Ag NPs on graphite electrode; Figure S3 EDX spectra recorded from the backside of the AAO/PANI/Ag sample.
