# Supplementary material for: 3D Nanocomposite with High Aspect Ratio Based on Polyaniline Decorated with Silver NPs: Synthesis and Application as Electrochemical Glucose Sensor
Source: Nanomaterials (Basel). 2023 Mar 10;13(6):1002. doi: 10.3390/nano13061002 (PMC10058674; doi:10.3390/nano13061002)
Supplement: Supplementary file 1 [file nanomaterials-13-01002-s001.zip › nanomaterials-2251470-supplementary.pdf]

An additional Teflon insert was made with fixing the counting electrode, which simultaneously served as a limiter of electrolyte volume. A photo of Teflon insert with linear dimensions is shown in Figure S1.

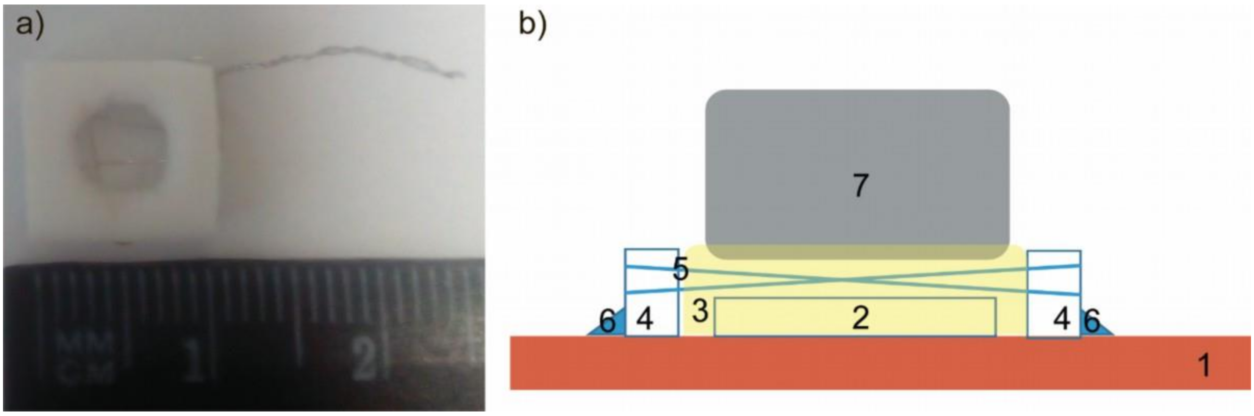


**Figure S1. (**a) photo of the detail with counting electrode; (b) scheme of electrochemical cell; 1 – copper plate, 2 – sample AAO/PANI/Ag, 3 – electrolyte, 4 – Teflon detail, 5 – counter electrode, 6 – holder, 7 – reference electrode.

The working electrode was prepared from AAO/PANI/Ag nanocomposite fixed on a copper plate. Then the part with the counting electrode was attached to the plate over the sample, the resulting cell was filled with blood plasma, the indicator electrode was immersed in the center of the cell.

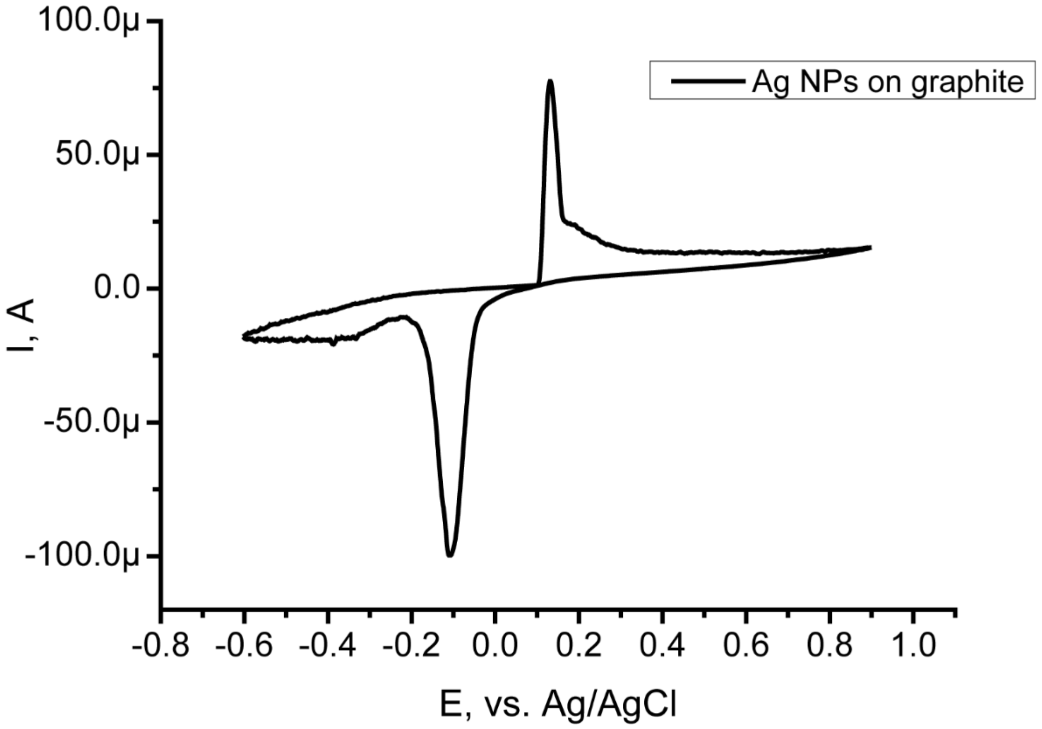


**Figure S2.** CV data for Ag NPs on graphite electrode. Measurements performed in Ringer's solution, the 3rd cycle presented.

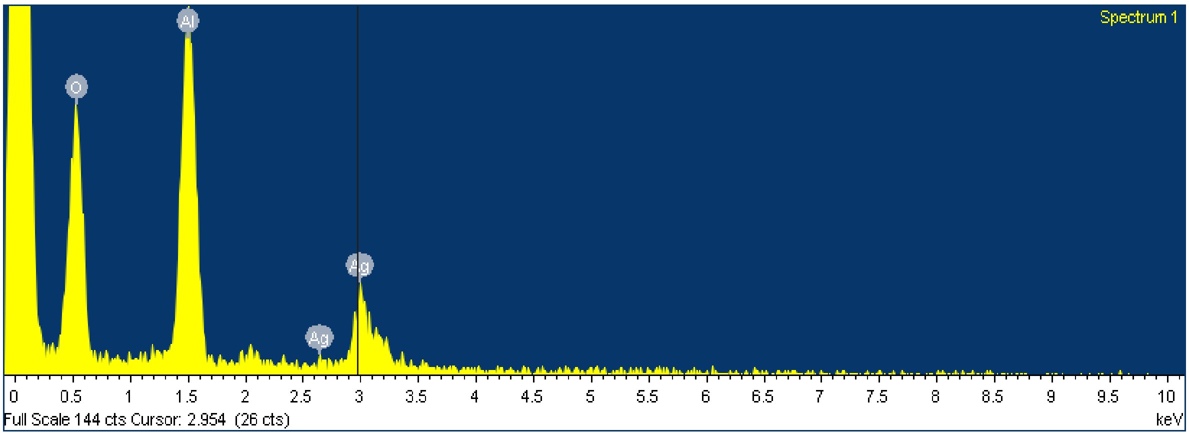


**Figure S3.** EDX spectra recorded from the backside of the AAO/PANI/Ag sample.
